# Fucoidan Derived from *Saccharina japonica* Modulates Intestinal Morphology and Gut Microbiota in Weaned Pigs

**DOI:** 10.3390/ani16162502

**Published:** 2026-08-11

**Authors:** Ping Liu, Peiru Li, Qixin Lin, Muyan Li, Jinbiao Zhao, Chuangxin Chen, Xiancheng Zeng, Qingqing Ding, Wenyi Jiang

**Affiliations:** 1 Fujian Key Laboratory of Traditional Chinese Veterinary Medicine and Animal Health, College of Animal Sciences, Fujian Agriculture and Forestry University, Fuzhou 350002, China; 2 College of Animal Sciences, Fujian Agriculture and Forestry University, Fuzhou 350002, China; 3 State Key Laboratory of Animal Nutrition, College of Animal Science and Technology, China Agricultural University, Beijing 100193, China; 4 Instrumental Analysis Center, Fujian Agriculture and Forestry University, Fuzhou 350002, China

**Keywords:** fucoidan, weaned piglets, intestinal morphology, gut microbiota, microbial metabolites

## Abstract

With global restrictions on in-feed antibiotics, sustainable alternatives are urgently needed to combat weaning-induced intestinal dysfunction in piglets. Fucoidan, a bioactive seaweed polysaccharide, exerts beneficial effects on gut health due to its immunomodulatory and prebiotic properties, but its mechanisms in the gastrointestinal tract of piglets remain unclear. This study investigated the effects of dietary fucoidan supplementation on growth performance, intestinal morphology, gut microbiota composition, and microbial metabolites in weaned piglets. The extracted fucoidan was characterized by a sulfate content of 23.7%, a molecular weight of 29.3 kDa, and fucose as the predominant monosaccharide. A total of 48 weaned piglets were randomly divided into two groups (*n* = 24 per group): a control group and a 500 mg/kg fucoidan treatment group. Growth performance was evaluated throughout the experiment. On day 28, jejunal and colonic tissues were collected to assess mucosal morphology (*n* = 6 per group), while cecal and colonic contents were collected for analysis of the gut microbiota and bacterial metabolites. Fucoidan supplementation significantly enhanced average daily gain (ADG, *p* < 0.05) and feed conversion ratio (FCR, *p* < 0.05) and mitigated diarrhea during the trial. Notably, piglets fed fucoidan exhibited increased villus height compared to controls (*p* < 0.05). Fucoidan modulated the gut microbiota by increasing cecal microbial diversity (*p* < 0.05) and shifting bacterial composition. These modifications involved an altered Bacteroidetes/Firmicutes ratio, as well as an enrichment of *Prevotellaceae*, *Lactobacillus*, and *Lachnospiraceae* (*p* < 0.05). Consistently, metabolite profiling analysis revealed increased cecal concentrations of lactate (*p* < 0.05) and butyrate (*p* < 0.05), which were positively associated with the growth of beneficial taxa. These findings demonstrate that fucoidan alleviates weaning-induced intestinal disorders by improving intestinal morphology and modulating gut microbiota and their metabolites. This study provides a strategy for utilizing marine-derived polysaccharides as functional feed additives to enhance animal health.

## Simple Summary

The global ban on antibiotics in animal feed has highlighted an urgent need for safe and sustainable alternatives. Weaning is a stressful period for young pigs, frequently causing intestinal disorders and poor growth. In this study, we investigated fucoidan derived from brown seaweed as a potential feed additive. We found that dietary fucoidan improved growth performance, reduced diarrhea incidence, and improved intestinal morphology in weaned pigs. Furthermore, it increased the relative abundance of specific gut bacteria and boosted the production of microbial metabolites that help modulate the mucosal immune response. The results demonstrated that fucoidan is a promising alternative to antibiotics that enhances intestinal health and reduces weaning stress in piglets. This study provides a novel strategy for the livestock industry to produce safe and high-quality animal products without relying on drugs, contributing to human health and environmental sustainability.

## 1. Introduction

Weaning stress is one of the major challenges in swine production. During this critical period, piglets are exposed to multiple stressors, such as maternal separation, social regrouping, environmental changes, and dietary transition. These stressors often compromise mucosal integrity and precipitate gut microbiota dysbiosis [1,2,3]. Such impairments significantly hinder nutrient digestion and absorption, resulting in post-weaning diarrhea, growth retardation, and substantial economic losses in the industry [4].

To mitigate these issues, antibiotic growth promoters (AGPs) and high-dose zinc oxide were widely used to prevent intestinal disorders and promote growth performance. However, prolonged use of these agents has contributed to antimicrobial resistance and environmental pollution, leading to their bans or restrictions in many countries [5,6]. Consequently, there is an urgent need to develop safe and effective alternatives to in-feed antibiotics for sustainable livestock production, such as probiotics, prebiotics, postbiotics, and plant extracts [7].

Among these promising alternatives, marine-derived bioactive compounds have garnered considerable attention for their unique pharmacological properties. *Saccharina japonica*, a widely cultivated brown seaweed, is particularly rich in fucoidan, a sulfated polysaccharide found primarily in the cell wall matrix [8]. Previous studies have shown that fucoidan exhibits a wide spectrum of biological activities, including antioxidant, anti-inflammatory, immunomodulatory, and prebiotic effects [9,10,11]. Indeed, supplementation with fucoidan has been shown to improve growth performance and reduce diarrhea in weaned piglets, potentially through modulating the abundance of beneficial and pathogenic bacteria and alleviating intestinal inflammation [12,13,14]. However, the bioactivity of fucoidan is highly dependent on its extraction source and purity, which vary widely among commercial products [15].

Gut microbiota and their metabolites play a pivotal role in maintaining intestinal homeostasis through crosstalk with the epithelial barrier and immune system [16,17]. It is hypothesized that the beneficial effects of polysaccharides are largely mediated by their ability to modulate the microbial ecosystem, which in turn promotes epithelial integrity. Therefore, this study aimed to investigate the effects of *Saccharina japonica*-derived fucoidan on intestinal morphology, gut microbiota composition, and metabolite profiles in weaned piglets. The findings will provide valuable insights into the application of fucoidan as a promising alternative to in-feed antibiotics for improving intestinal health in weaned piglets.

## 2. Materials and Methods

### 2.1. Extraction and Purification of Fucoidan Derived from Saccharina japonica

Fresh *Saccharina japonica* was harvested from Ningde, Fujian Province, a major seaweed cultivation region in China. The extraction and purification procedures were performed based on previously described methods [18], with slight modifications. Briefly, the brown seaweed was rinsed and pulverized to pass through a 300-mesh sieve. Lipids were removed by refluxing the powder with 90% ethanol, and the residue was extracted with ultrapure water (1:50, *w*/*v*) at 95 °C for 8 h. After concentration, alginate was eliminated by precipitation with 0.15 M CaCl_2_, and the supernatant was collected. Crude fucoidan was precipitated by adding three volumes of 95% ethanol, and proteins were removed using the Sevage method. The crude polysaccharide was then dissolved, filtered, and sequentially purified by DEAE-cellulose-52 chromatography (Cytiva, Marlborough, MA, USA) eluted with 0.8 M NaCl, followed by Sephadex G-100 gel filtration (Cytiva, Marlborough, MA, USA). Finally, the purified fraction was dialyzed using a 1000 Da molecular weight cutoff (MWCO) membrane.

### 2.2. Chemical Composition Analysis of Fucoidan

The total sugar concentration of extracted fucoidan was quantified using the phenol-sulfuric acid method [19], while sulfate group content was determined via the barium chloride-gelatin turbidimetric method [20]. Monosaccharide composition analysis was performed by hydrolyzing fucoidan with trifluoroacetic acid, followed by derivatization with 1-phenyl-3-methyl-5-pyrazolone (PMP) and subsequent analysis using HPLC (E2695, Waters, Milford, MA, USA), according to the method described by Li et al. [18], with minor modifications. HPLC conditions were as follows: the chromatographic column was the ZORBAX Eclipse XDB-C18, with a mobile phase consisting of acetonitrile and phosphate buffer (18:82, pH 6.7), a flow rate of 1 mL/min, a column temperature of 30 °C, and a detection wavelength of 254 nm. Monosaccharides quantified included fucose, mannose, rhamnose, glucose, arabinose, xylose, ribose, and galactose. Molecular weight was determined by high-performance gel permeation chromatography (1260 Infinity II GPC/SEC, Agilent Technologies, Santa Clara, CA, USA) using PL aquagel–OH columns. The eluent was 0.1 M sodium nitrate at a flow rate of 1 mL/min, a column temperature of 45 °C, and an injection volume of 20 µL of dissolved samples.

### 2.3. Animal Experiments, Diet Treatments, and Sampling

All procedures involving pigs were approved by the Institutional Animal Care and Use Committee of Fujian Agriculture and Forestry University (FZCASFAFU21041) and complied with the Laboratory Animal Welfare and Ethics Guideline of China (ICS 65.020.30).

Forty-eight weaned Duroc × (Landrace × Yorkshire) piglets (28 ± 1 days) with a mean body weight (BW) of 7.43 ± 0.08 kg were randomly assigned to two groups (*n* = 24/group) balanced for litter and gender. Following group allocation, the initial body weights of the animals across the groups were compared using an independent-sample *t*-test. The results indicated no statistically significant differences between the groups (*p* > 0.05). Piglets were housed in commercial flat-deck pens (4 piglets/pen, 6 pens per group) under controlled conditions: room temperature was maintained at 26 °C on weaning day and gradually reduced to 22 °C by day, with humidity at 65–75%. Feed and water were provided ad libitum to meet the pigs’ physiological requirements and ensure animal welfare. The experimental diets in this study were formulated in accordance with the nutrient requirements of swine recommended by NRC (2012). Both groups were fed fiber-free experimental diets (Table 1) based on corn starch and isolated soybean protein: a control diet and a fucoidan-supplemented diet (500 mg/kg fucoidan). This supplementation level was based on a previous study by Huang et al. [21], which demonstrated that this level promoted intestinal homeostasis. After a 7-day acclimation, the trial lasted 28 days. At the end of the trial, one piglet was randomly selected from each pen for slaughter and sampling (*n* = 6/per group). Furthermore, the sampling process was controlled to ensure an equal sex ratio in each group (3 males and 3 females). Piglets were euthanized with ketamine hydrochloride (15 mg/kg BW; Jiangsu Zhongmu Beikang Pharmaceutical Co., Ltd., Taizhou, China) and propofol (3.5 mg/kg BW; Xi′an Libang Pharmaceutical Co., Ltd., Xi′an, China), followed by euthanasia via intracardial injection of potassium chloride (50 mg/kg BW; Jilin Huamu Animal Health Products Co., Ltd., Changchun, China). The entire gut was excised, and the small intestine was separated from the large intestine and mesentery. A 2 cm segment of the jejunum and colon was fixed in Carnoy solution for histological analysis. Cecal and colonic digesta were collected, snap-frozen in liquid nitrogen, and stored at −80 °C for gut microbiota composition and organic acid analysis.

### 2.4. Evaluation of Growth Performance and Fecal Score During the Weaning Period

The amount of feed provided to weaned piglets was recorded, and any unconsumed feed was weighed daily to calculate the average daily feed intake (ADFI). Piglets were weighed individually on days 0, 14, and 28 throughout the entire experimental period. Average daily gain (ADG) was calculated as the total weight gain (final body weight − initial body weight) divided by the number of treatment days. The feed conversion ratio (FCR) was determined as the ratio of ADFI to ADG. Diarrhea incidence was monitored throughout the study, and fecal consistency was scored according to the method described by Pierce et al. [22]. The scoring criteria were as follows: 0 = no feces, 1 = hard, firm feces, 2 = pasty feces, 3 = droplets of watery feces/diarrhea, 4 = moderate diarrhea, and 5 = severe diarrhea.

### 2.5. Intestinal Histomorphological Analysis

Fresh jejunal and colonic tissues were immediately fixed in Carnoy’s solution (methanol–chloroform–glacial acetic acid = 6:3:1, *v*/*v*) at 4 °C for 2 h, followed by dehydration in a graded ethanol series. Tissues were embedded in paraffin, and 5 µm sections were cut using a microtome. Sections were dried at 37 °C, deparaffinized in xylene, and rehydrated through a descending ethanol series. Intestinal tissues were stained with hematoxylin and eosin (H&E). Morphological evaluation was performed using a Nikon ECLIPSE TS2-FL inverted fluorescence microscope (Nikon, Tokyo, Japan). Jejunal and colonic morphological analysis included villus height, crypt depth, and the villus height-to-crypt depth ratio. Ten well-oriented villi and their corresponding crypts were randomly measured in each piglet.

### 2.6. 16S rRNA Gene Sequencing

Microbial genomic DNA was isolated from the cecal and colonic digesta using an E.Z.N.A. Stool DNA Kit (Omega Bio-tek, Norcross, GA, USA). We evaluated DNA integrity and purity via 1% agarose gel electrophoresis and measured concentration using a NanoDrop spectrophotometer (Thermo Scientific, Wilmington, DE, USA). To characterize the bacterial community, the V3-V4 hypervariable region of the 16S rRNA gene was amplified using the primers 338F (5′-barcode-ACTCCTACGGGAGGCAGCAG-3′) and 806R (5′-GGACTACHVGGGTWTCTAAT-3′) on an ABI GeneAmp 9700 thermocycler (Applied Biosystems, Foster City, CA, USA). The PCR mixture (20 μL total volume) consisted of 4 μL of 5× TransStart FastPfu buffer, 2 μL of 2.5 mM dNTPs, 0.8 μL of each primer (5 μM), 0.4 μL of TransStart FastPfu polymerase, and 10 ng of template DNA. The final amplicons were resolved on a 2% agarose gel, recovered with the AxyPrep DNA Gel Extraction Kit (Axygen Biosciences, Union City, CA, USA), and quantified with a Quantus™ Fluorometer (Promega, Madison, WI, USA). Purified PCR products were mixed in equimolar ratios and sequenced on an Illumina MiSeq PE300 platform (Illumina, San Diego, CA, USA).

The obtained raw reads were demultiplexed and filtered for quality using fastp (v20.0), followed by merging with FLASH (v1.2.7). Sequences were clustered into operational taxonomic units (OTUs) at a 97% similarity threshold using UPARSE (v7.1), during which chimeric sequences were identified and discarded via UCHIME. Taxonomic classification of OTU representative sequences was performed by the RDP Classifier (v2.2) against the SILVA database with an 80% confidence cutoff. Microbial diversity within the samples (α-diversity) was estimated using the Chao1 and Shannon indices, while inter-sample variation (β-diversity) was visualized using principal component analysis (PCA). Microbial community composition was visualized using bar charts at the phylum and genus levels. Differentially abundant taxa were identified by the Wilcoxon rank-sum test (*p* < 0.05) with linear discriminant analysis effect size (LEfSe). The raw sequencing data have been deposited in the NCBI Sequence Read Archive (SRA) under accession number PRJNA 1293694.

### 2.7. Quantification of Organic Acids

Cecal and colonic digesta (0.5 g each) were homogenized in 8 mL of ultrapure water for 30 min using an ultrasonic bath, followed by centrifugation at 8000× *g* for 10 min. The suspension was diluted 1:50 with water and filtered through a 0.22 μm membrane. A 25 µL aliquot of the filtered sample was analyzed for the organic acids, including lactate, acetate, propionate, and butyrate, using high-performance ion chromatography (Dionex ICS-3000, Thermo Fisher Scientific, Sunnyvale, CA, USA). Separation was performed using an AS11 analytical column (250 mm × 4 mm) and an AG11 guard column. A potassium hydroxide gradient was applied under the following conditions: 0–5 min, 0.8–1.5 mM; 5–10 min, 1.5–2.5 mM; 10–15 min, 2.5 mM, with a constant flow rate of 1.0 mL/min.

### 2.8. Statistical Analysis

Data were analyzed using Prism 6 software (GraphPad Software, La Jolla, CA, USA). Differences between the two dietary treatments were assessed using a two-tailed Student’s *t*-test. Sample sizes were determined based on the preliminary experiments to ensure adequate statistical power. Data are presented as means ± SEM. Significance levels are denoted as * *p* < 0.05 and ** *p* < 0.01.

## 3. Results

### 3.1. Chemical Composition and Characterization of Fucoidan

It is well established that the structural and chemical properties of natural polysaccharides are closely linked to their biological activities. In this study, the extracted fucoidan had a total sugar content of 75.7%, with sulfate and uronic acid contents of 23.7% and 6.5%, respectively (Table 2). The average molecular weight of the samples was 29.3 kDa. Monosaccharide composition analysis revealed that the polysaccharide consisted primarily of fucose (75.46%), galactose (22.04%), and mannose (1.03%).

### 3.2. Dietary Fucoidan Improved Growth Performance

**Figure 1:**
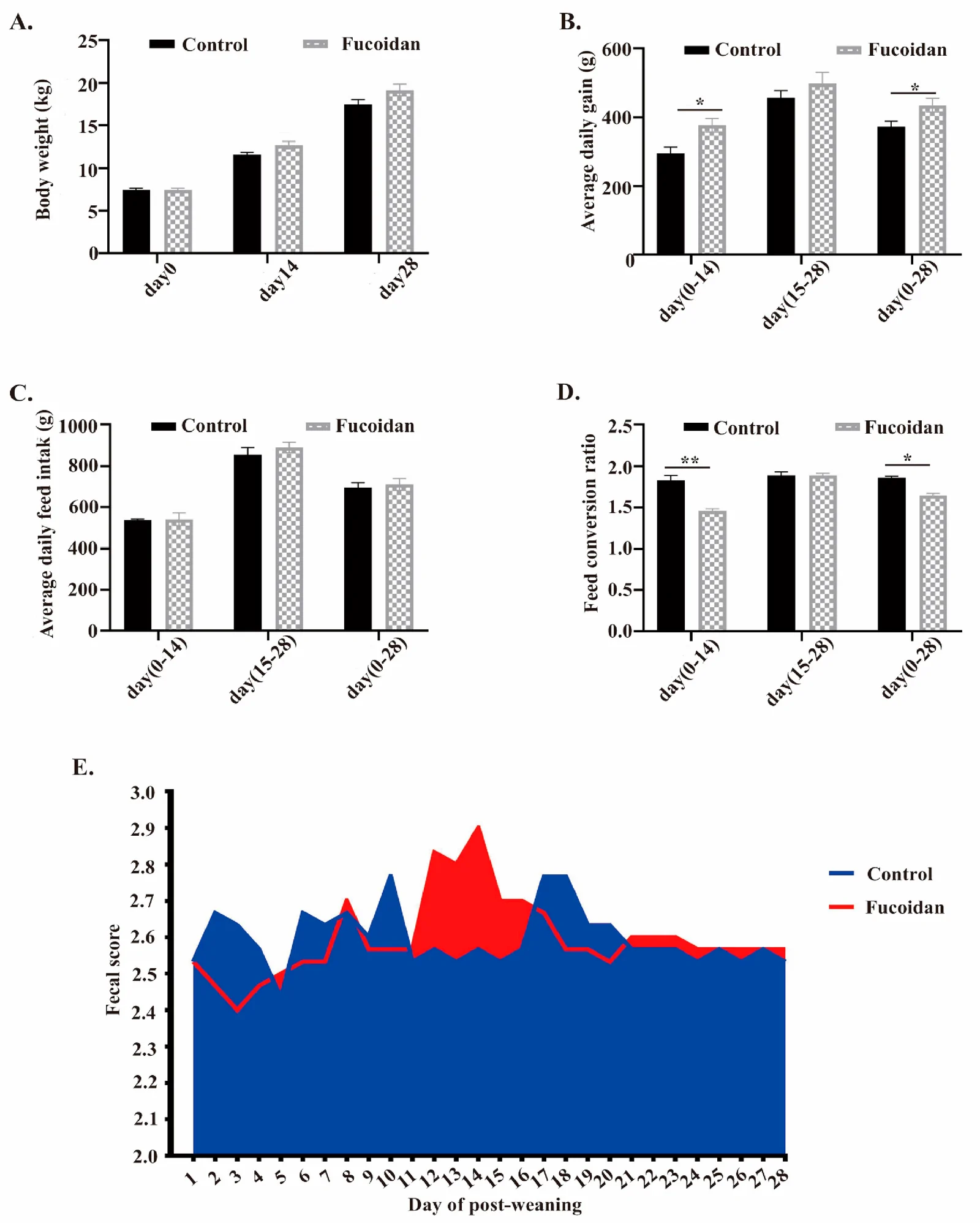
Effects of dietary fucoidan on growth performance in weaned piglets: (**A**) Body weight. (**B**) Average daily gain. (**C**) Average daily feed intake. (**D**) Feed conversion ratio. (**E**) Fecal scores of piglets were evaluated throughout the experimental period. * *p* < 0.05, ** *p* < 0.01, *n* = 24 per group.

During the first 14 days post-weaning, although BW and ADFI did not differ significantly, piglets receiving fucoidan exhibited a significantly higher ADG (Figure 1A–C, *p* < 0.05). The fucoidan group also demonstrated a significantly lower FCR than the control group (Figure 1D, *p* < 0.01). From day 15 to day 28, growth performance was comparable between the two groups. Over the entire 28-day trial period, piglets fed fucoidan showed increased ADG (Figure 1B, *p* < 0.05) and improved FCR compared to controls (Figure 1D, *p* < 0.05).

Fecal consistency was assessed daily using a standardized scoring system. Piglets supplemented with fucoidan displayed no clinical signs of diarrhea throughout the study. Their fecal scores generally remained lower and more stable than those of the control group, although a transient fluctuation was observed between days 11 and 17 (Figure 1E).

### 3.3. Dietary Fucoidan Enhanced Intestinal Morphology

**Figure 2:**
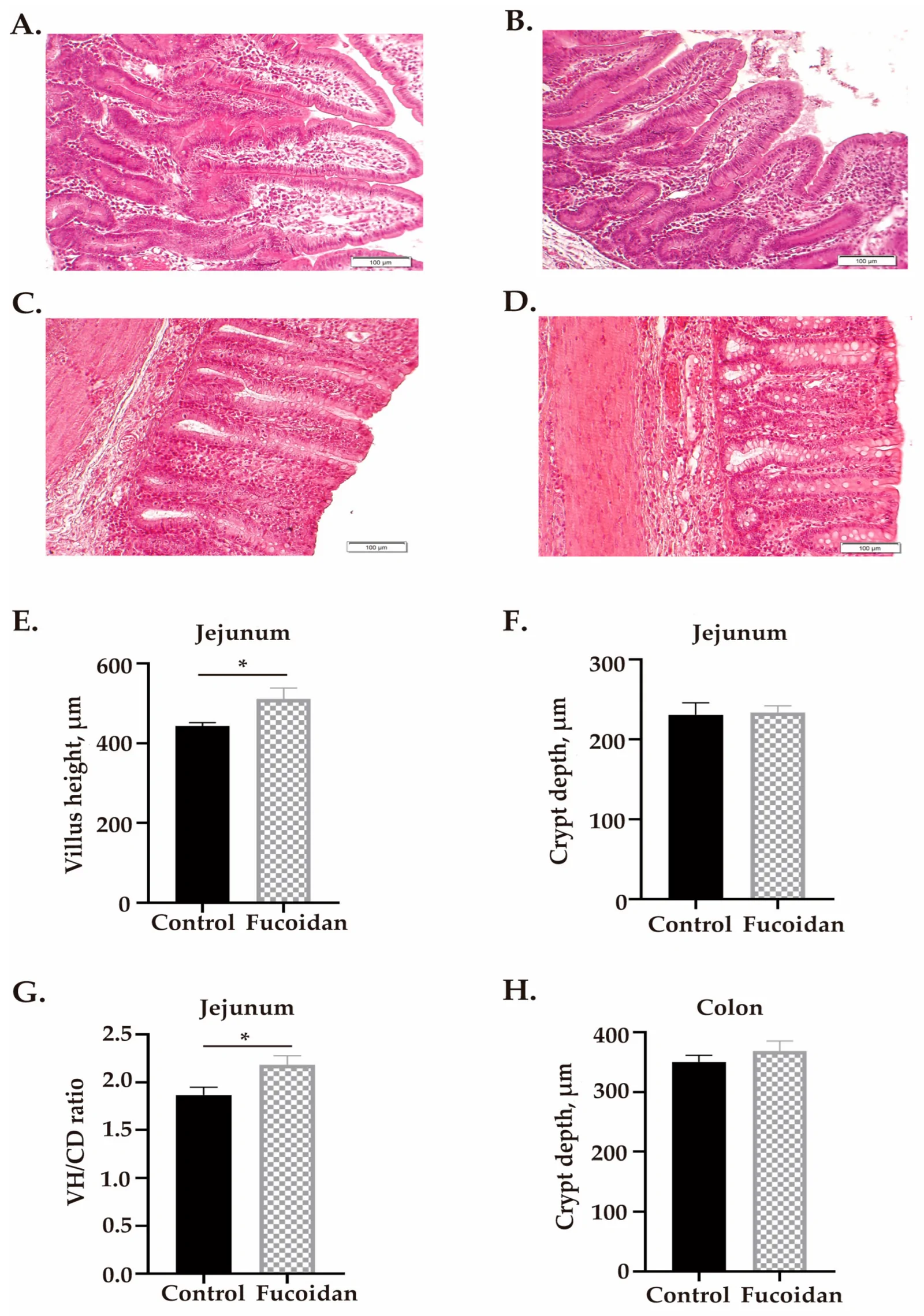
Effects of dietary fucoidan on intestinal morphology in weaned piglets: (**A**) Jejunal morphology in the control group (100×). (**B**) Jejunal morphology in the fucoidan treatment group (100×). (**C**) Colonic morphology in the control group (100×). (**D**) Colonic morphology in the fucoidan treatment group (100×). (**E**) Villus height in the jejunum. (**F**) Crypt depth in the jejunum. (**G**) Villus height-to-crypt depth ratio in the jejunum. (**H**) Crypt depth in the colon. * *p* < 0.05, *n* = 6 per group.

Given the close relationship between intestinal morphology and animal health, histomorphometric analysis was performed (Figure 2A–D). The results showed that the piglets fed fucoidan exhibited significantly higher villus height (Figure 2E, *p* < 0.05) and an increased villus height-to-crypt depth ratio compared to the control group (Figure 2G, *p* < 0.05). In contrast, crypt depth showed no significant differences between the two treatment groups in either the jejunum (Figure 2F) or colon (Figure 2H).

### 3.4. Dietary Fucoidan Supplementation Altered Gut Microbiota Composition

**Figure 3:**
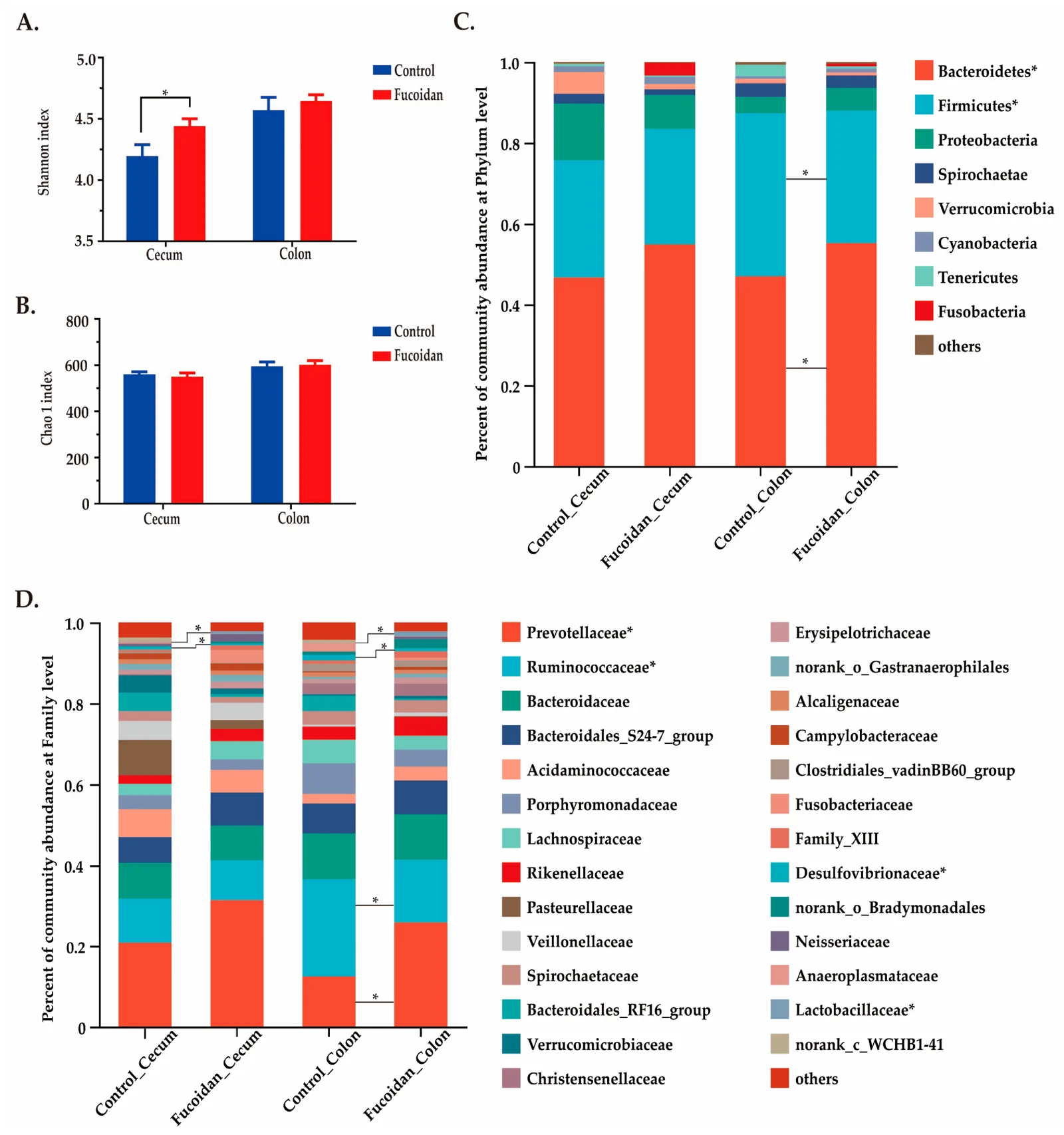
Dietary fucoidan altered gut microbiota composition in weaned piglets: (**A**) Shannon index of α-diversity. (**B**) Chao1 index of α-diversity. (**C**) Gut microbiota composition at the phylum level. (**D**) Gut microbiota composition at the family level. * *p* < 0.05, *n* = 6 per group.

The intestinal microbiota profiles of piglets were characterized using 16S rRNA gene amplicon sequencing. Shannon and Chao1 indices, representing bacterial α-diversity, were calculated (Figure 3A,B). Compared with the control group, piglets receiving a fucoidan-supplemented diet showed a significantly higher Shannon index in the cecum (*p* < 0.05), indicating enhanced microbial diversity. However, the Chao1 index showed no significant differences between the fucoidan and control groups in either the cecum or the colon (*p* > 0.05). This indicates that dietary intervention did not significantly alter the species richness of the gut microbiota. The dominant phylum in the large intestine was Bacteroidetes (46.8% of the microbiota), followed by Firmicutes (34.7%) and Proteobacteria (9.0%), which may be associated with the relatively low fiber content of the basal diet. In the colon, fucoidan intervention significantly increased the relative abundance of Bacteroidetes (Figure 3C, *p* < 0.05) and decreased that of Firmicutes (*p* < 0.05).

At the family level, *Prevotellaceae* was the most abundant taxon in the large intestine. Fucoidan supplementation significantly increased the relative abundance of this family in colonic contents compared with the control group (Figure 3D, *p* < 0.05). In contrast, the abundance of *Ruminococcaceae* was markedly reduced in the colon of the fucoidan group (*p* < 0.05). Additionally, the abundance of *Desulfovibrionaceae* decreased significantly in both the cecal and colonic contents of fucoidan-fed piglets (*p* < 0.05), whereas the abundance of *Lactobacillaceae* increased in both gut segments (*p* < 0.05).

**Figure 4:**
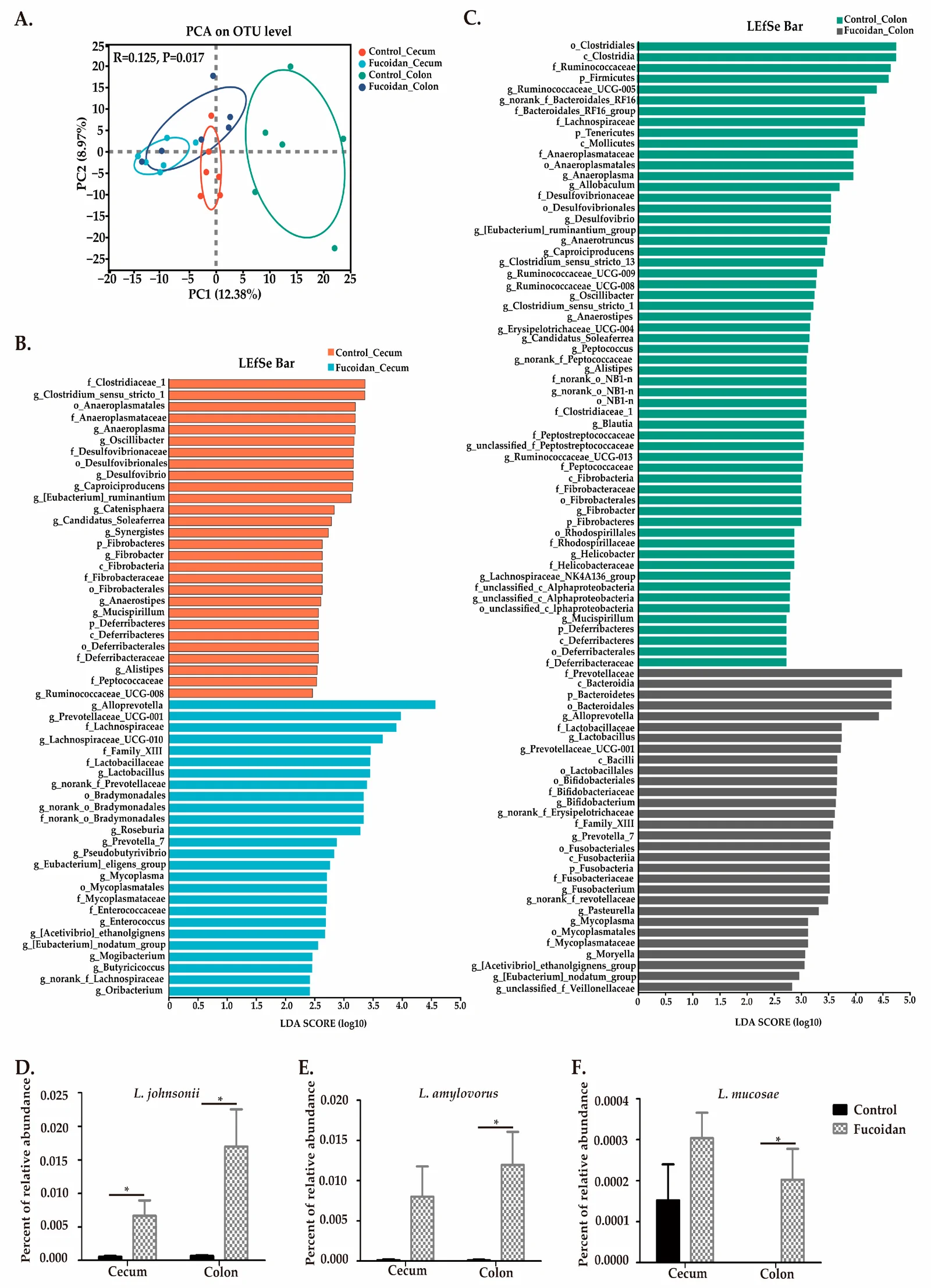
Effects of dietary fucoidan on the abundance of gut microbes with significant changes: (**A**) PCA of microbial diversity. (**B**) LEfSe analysis of differentially abundant bacterial communities in the cecum. (**C**) LEfSe analysis of differentially abundant bacterial communities in the colon. (**D**) Relative abundance of *L. johnsonii*. (**E**) Relative abundance of *L. amylovorus*. (**F**) Relative abundance of *L. mucosae*. * *p* < 0.05, *n* = 6 per group.

Principal component analysis (PCA) showed a clear separation between the bacterial communities of control and fucoidan-treated piglets in both cecal and colonic digesta (Figure 4A, *p* < 0.05). In the cecum, linear discriminant analysis effect size (LEfSe) identified significant enrichment of several taxa belonging to Bacteroidetes in the fucoidan group, including *Alloprevotella*, *Prevotellaceae_UCG-001*, and *Prevotella_7* (Figure 4B). Within the Firmicutes phylum, enriched taxa included genera such as *Lachnospiraceae_UCG-010*, *Lactobacillus*, *Roseburia*, *Pseudobutyrivibrio*, *Eubacterium eligens*, *Eubacterium nodatum*, and *Butyricicoccus*.

Further LEfSe analysis highlighted distinct microbial shifts in the colon (Figure 4C). The fucoidan group exhibited increased abundance of the *Bacteroidetes* taxa, such as *Alloprevotella*, *Prevotellaceae_UCG-001*, and *Prevotella_7*, along with enrichment of *Firmicutes*-affiliated taxa, including *Lactobacillus* and *Acetivibrio ethanoligenes*. Additionally, a significant increase in the *Actinobacteria* genus *Bifidobacterium* was also observed.

Notably, the relative abundance of *Lactobacillus johnsonii* was significantly higher in both the cecal and colonic microbiota of fucoidan-treated piglets (Figure 4D, *p* < 0.05). In the colon, the abundance of *L. amylovorus* and *L. mucosae* was also elevated (Figure 4E,F, *p* < 0.05).

### 3.5. Dietary Fucoidan-Modulated Bacterial Metabolites

**Figure 5:**
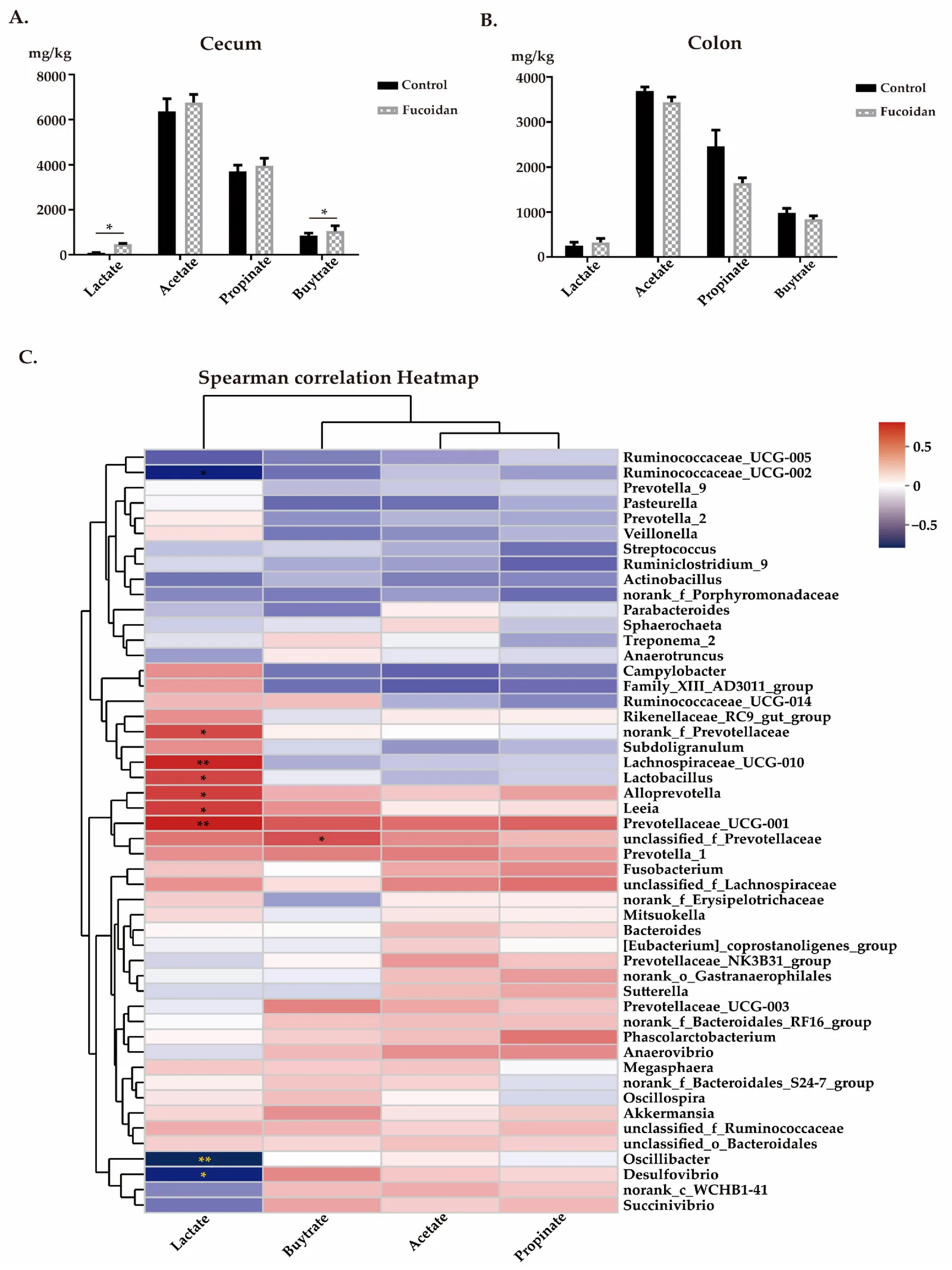
Influence of dietary fucoidan on bacterial metabolites in the large intestine of piglets: (**A**) Bacterial metabolites in the cecum. (**B**) Bacterial metabolites in the colon. (**C**) Correlation analysis between differentially abundant gut microbes and metabolites in the cecum. * *p* < 0.05, ** *p* < 0.01, *n* = 6 per group.

Short-chain fatty acids (SCFAs) and lactate are key metabolites derived from bacterial fermentation of natural polysaccharides. SCFAs primarily include acetate, propionate, and butyrate, while lactate serves as another important fermentation product in the gut. In the cecum, piglets fed a fucoidan-supplemented diet exhibited notable alterations in metabolite profiles. Lactate levels were five times higher than those in the control group (Figure 5A, *p* < 0.05). This accumulated lactate likely served as a metabolic substrate for butyrate-producing bacteria, contributing to the subsequent significant increase in butyrate content compared with the control (Figure 5A, *p* < 0.05). In contrast, colonic concentrations of lactate, acetate, propionate, and butyrate showed no significant changes (Figure 5B, *p >* 0.05). These regional differences in SCFAs and lactate profiles suggested that fucoidan is preferentially fermented in the cecum rather than the colon, likely due to variations in microbial composition and microenvironmental conditions between the two intestinal segments.

Furthermore, correlation analysis was performed to evaluate the associations between the genus-level cecal microbiota and the levels of lactate, acetate, propionate, and butyrate. The analysis revealed that lactate was positively correlated with *norank_f_Prevotellaceae*, *Lachnospiraceae_UCG_010*, *Lactobacillus*, *Alloprevotella*, *Leeia*, and *Prevotellaceae_UCG_001* but negatively correlated with *Oscillibacter* and *Desulfovibrio*. Additionally, butyrate was positively associated with *Prevotellaceae* (Figure 5C). The concurrent increase in lactate and butyrate suggests a potential synergistic interaction within the cecal microbial community under fucoidan intervention.

## 4. Discussion

Fucoidan, a sulfated polysaccharide derived from brown algae, possesses a highly branched structure composed of fucose, mannose, xylose, galactose, and sulfate groups [10]. These structural attributes, particularly high fucose content and degree of sulfation, are fundamental to its multifunctional roles in host–microbe interactions and immune regulation. Specifically, fucose mediates specific crosstalk between epithelial cells and gut microbiota [23]. Furthermore, sulfate groups enhance water solubility and contribute to immune modulation by inhibiting pathogen adhesion and activating immune cells [9,11]. Minor monosaccharides such as galactose and mannose form branched chains that may act synergistically to enhance bioactivity, aligning with the complex glycan profiles typical of brown algal fucoidans.

In the context of the global ban on AGPs in livestock production, seaweed-derived compounds such as fucoidan have emerged as promising antibiotic alternatives [24,25]. In the present study, a dietary fucoidan supplementation level of 500 mg/kg was selected. This dosage was based on previous evidence that this concentration effectively promotes intestinal homeostasis [21]. Importantly, toxicological evaluations support the safety of this dosage; studies have shown that fucoidan is safe for oral consumption at doses up to 1000 mg/kg body weight per day in rats, with no signs of toxicity in biochemical or hematological analyses [26]. Since the estimated intake in our animals is far below this established safety threshold, the selected dosage ensures both physiological efficacy and safety for the experimental animals. Previous studies have shown that seaweed bioactives can alleviate weaning stress. For example, dietary supplementation with *Ecklonia cava* has been shown to improve ADG in weanling pigs [27]. Similarly, combinations of fucoidan and laminarin enhanced growth performance [13]. Consistent with these findings, our results demonstrated that fucoidan supplementation significantly enhanced ADG and feed utilization efficiency, suggesting that fucoidan may alleviate weaning stress in piglets. Despite the improved growth performance, a transient fluctuation in fecal scores was observed between days 11 and 17; this likely reflects an adaptive reconstruction of the gut microbiota. Fucoidan is a soluble non-starch polysaccharide that serves as a fermentable substrate for gut microbes. In the initial stage (days 1–10), fucoidan likely exerted protective effects through its physicochemical properties or by binding to extracellular receptors to regulate the intestinal barrier function [28]. However, as the gut microbiota gradually re-established stability post-weaning (days 11–17), microbial utilization and fermentation of fucoidan intensified. This process was accompanied by shifts in microbial community structure and fluctuations in metabolites, which may have temporarily altered the intestinal osmotic pressure or the physical properties of the digesta, resulting in transiently looser feces [3,29]. Subsequently, as the host adapted to the dietary change and a new microbial homeostasis was established, the fermentation pattern stabilized, and fecal consistency returned to normal. Therefore, the fluctuation observed during days 11–17 was not a pathological onset of severe diarrhea but rather a dynamic adaptation process of the gut microbiota in response to dietary intervention.

To further elucidate the physiological basis underlying these performance improvements, the intestinal morphology of the piglets was investigated. We chose the jejunum for the following two reasons: First, the jejunum is a primary site for nutrient digestion and absorption. Second, the jejunum in piglets is relatively long and rich in lymphoid tissues (such as Peyer’s patches), which contain abundant antigen-presenting cells, including dendritic cells. As a sulfated polysaccharide, fucoidan can regulate innate immune responses by activating receptors on immune cells and epithelial cells, thereby exerting a beneficial effect on intestinal barrier function [30]. Meanwhile, the colon harbors approximately 90% of the gut microbiota, and the degradation and utilization of fucoidan are highly dependent on the participation of these microorganisms. Furthermore, fucoidan is fermented by gut microbes in the colon to produce metabolites such as SCFAs, which play a significant role in regulating colon development and function [31]. Therefore, these two regions were investigated to determine the major effects of fucoidan on intestinal morphology in the present study. It is well established that weaning stress typically induces villus atrophy and crypt hyperplasia, leading to a reduced digestive and absorptive capacity [2,3]. Our results demonstrated that fucoidan significantly enhanced villus height in the jejunum and mitigated these detrimental structural changes. The increase in jejunal villus height directly expands the surface area available for nutrient absorption, which provides a direct explanation for the improved ADG and feed efficiency observed throughout the 28-day trial. In addition, the increased villus height-to-crypt depth ratio suggests a more mature and functional intestinal epithelium, implying that the gut was in a state of active absorption rather than of weaning-stress-induced inflammatory pathology. The beneficial effects of fucoidan on intestinal morphology may be associated with its anti-inflammatory and prebiotic properties [10,31].

Fucoidan resists endogenous hydrolysis in the upper gastrointestinal tract and is fermented in the lower intestine by specific commensal microbiota [32,33]. Previous studies have shown that fucoidan can increase the abundance of beneficial bacteria (e.g., *Lactobacillus* and *Bifidobacterium*) and inhibit the growth of pathogenic bacteria (e.g., *Escherichia coli* and *Salmonella typhimurium*) in the intestinal tract of weaned piglets [13,14,25]. However, most studies are limited to quantitative analysis of specific bacterial genera, and a comprehensive understanding of the shifts in the overall structure of the intestinal microbiota remains elusive. In addition, conventional corn–soybean basal diets inherently contain approximately 12% total dietary fiber [34]. Consequently, the content of this background fiber far exceeds the amount of fucoidan supplemented in the present study. Therefore, a low-fiber purified diet formulated with corn starch and soybean protein isolate was specifically used to eliminate dietary fiber interference [35,36,37] and clarify the key prebiotic function of fucoidan. The results revealed a significant increase in microbial diversity of the large intestine, characterized by an enrichment of Bacteroidetes and specific proliferation of *Prevotella* and *Lactobacillus*. As a keystone phylum for degrading complex carbohydrates, the enrichment of Bacteroidetes suggests an enhanced capacity for polysaccharide breakdown and subsequent fermentation in the gut ecosystem [38,39]. Notably, the marked increase in *Prevotella* indicates selective enrichment of strains harboring specialized enzymes for fucoidan degradation, such as sulfatases and α-L-fucosidases, in the porcine gut [40,41].

*Lactobacillus* exerts antibacterial effects by secreting bacteriocins and competitively excluding pathogens to prevent bacterial adhesion [42]. The increased abundance of *L. johnsonii*, *L. amylovorus*, and *L. mucosae* in piglets supplemented with dietary fucoidan was consistent with previous studies [10,13,43] and may explain the reduced incidence of diarrhea. Additionally, a decrease in *Desulfovibrionaceae* should help mitigate intestinal inflammatory damage by reducing hydrogen sulfide production [44]. LEfSe analysis demonstrated that fucoidan intervention significantly enriched specific taxa, such as *Alloprevotella*, *Prevotella_7*, and *Bifidobacterium*. This microbial profile suggests the formation of a protective microbiota-immune axis. Specifically, *Alloprevotella* and *Prevotella* are known to be involved in mucin metabolism, which could help maintain the intestinal mucus barrier [45]. Furthermore, *Bifidobacterium* enrichment may contribute to immunomodulation by promoting anti-inflammatory cytokines [46].

Fucoidan intervention induced a significant shift in the gut metabolic network of piglets. Correlation analysis highlighted specific microbial functions. Firstly, *Lactobacillus* is well-recognized for its ability to produce lactic acid [42]. Consequently, its significant enrichment resulted in a marked accumulation of lactate, providing a key fermentation substrate. Secondly, the family *Prevotellaceae* also showed a strong positive correlation with lactate levels, suggesting its active involvement in fucoidan degradation and the broader metabolic context supporting lactate accumulation. This metabolic activity could supply precursors as a co-substrate that further facilitates the fermentation process [47]. Moreover, the abundance of *Lachnospiraceae* as key butyrate producers was significantly positively correlated with lactate levels as well. This indicated that lactate generated by the upstream bacteria was efficiently utilized by *Lachnospiraceae* via the cross-feeding networks to produce butyrate [47,48]. Ultimately, this synergistic interaction explains the significant elevation of butyrate in the intestine. Butyrate is the preferred energy source for colonocytes and plays critical roles in maintaining intestinal homeostasis [49,50], and its elevation is closely associated with enhanced intestinal morphology and growth performance. Specifically, the significantly elevated levels of lactate and butyrate demonstrate that the fucoidan activated microbial metabolic activity in the cecum, the primary site of fermentation, marking the initiation of the hindgut response to the intervention. As the bacterial metabolites diffused into the colon along with the digesta, they were rapidly absorbed by colonic epithelial cells and immune cells to maintain mucosal barrier function, thereby reshaping the gut microenvironment to favor host–microbe symbiosis. Concurrently, the continuous influx of fucoidan fermentation products altered the local nutritional niche in the colon, driving the succession of dominant bacterial taxa toward groups adapted to utilize these specific substrates, which ultimately led to a significant remodeling of the colonic microbial composition.

Although this study demonstrated the beneficial effects of fucoidan on gut microbiota and intestinal morphology, the underlying mechanisms remain not fully elucidated. While existing data have revealed alterations in specific bacterial taxa and metabolites, the lack of functional validation precludes the identification of key factors directly mediating these effects, and the relevant signaling pathways have not been explored. Future research should focus on identifying key functional microbes and molecular mechanisms through fecal microbiota transplantation and gene knockout experiments, thereby providing a deeper insight into the mechanisms by which fucoidan maintains intestinal health in pigs.

## 5. Conclusions

This study demonstrates that dietary fucoidan improves growth performance and intestinal morphology in weaned piglets, possibly through modulation of the gut microbiota, specifically *Lactobacillus*, *Prevotella*, and *Lachnospiraceae* populations, as well as their metabolic products of lactate and butyrate. Thus, seaweed-derived fucoidan represents a promising strategy to enhance intestinal health and mitigate post-weaning stress.

## Figures and Tables

**Table 1:** The composition of experimental diets and nutritional values (as-fed basis).

Ingredient	Control Treatment	Fucoidan Treatment
Corn starch	59.72	59.67
Soybean protein isolate	12.80	13.00
Fish meal	10.00	9.80
Whey powder	8.00	8.00
Soybean oil	0.45	0.45
Sucrose	2.00	2.00
Dextrose	4.00	4.00
Flavor powder	0.05	0.05
Sweetener	0.03	0.03
Fucoidan	–	0.05
Dicalcium phosphate	1.40	1.40
Limestone	0.20	0.20
Sodium chloride	0.15	0.15
Lysine-HCl	0.35	0.35
D, L-Methionine	0.10	0.10
L-Threonine	0.20	0.20
Tryptophan	0.05	0.05
Vitamin-mineral premix ^1^	0.50	0.50
Total	100	100
Nutritional value ^2^		
Digestible energy, Kcal/kg	3542	3543
Crude protein, %	18.41	18.45
Calcium, %	0.84	0.83
Total phosphorus, %	0.65	0.65
SID Lysine, %	1.39	1.39
SID Methionine, %	0.40	0.40
SID Threonine, %	0.81	0.81
SID Tryptophan, %	0.25	0.25

^1^ Vitamin and mineral premix provides the following per kilogram of diet: 15,000 IU vitamin A; 8000 IU vitamin D3; 30 IU vitamin E; 2.5 mg vitamin K3; 2.5 mg vitamin B1; 4.0 mg vitamin B2; 3.0 mg vitamin B6; 20 μg vitamin B12; 40 mg nicotinic acid; 12.5 mg pantothenic acid; 0.7 mg folic acid; 20 μg biotin; 400 mg Fe as ferrous sulfate; 150 mg Cu as basic cupric chloride; 150 mg Zn as zinc sulfate; 80 mg Mn as manganese sulfate; 0.4 mg I as ethylenediamine dihydroiodide; 0.3 mg Se as sodium selenite. ^2^ Nutritional levels were calculated values.

**Table 2:** The molecular weight and chemical analysis of fucoidan.

Item	Content
Total sugar	75.7, %
Sulfate content	23.7, %
M_w_ ^1^	29.3, kDa
M_w_/M_n_ ^2^	1.25
Fucose	75.46, %
Galactose	22.04, %
Mannose	1.03, %
Glucose	0.53, %
Arabinose	0.29, %
Xylose	0.28, %
Rhamnose	0.25, %
Ribose	0.12, %

^1^ M_w:_ weight-average molecular weight. ^2^ M_w_/M_n_ = weight-average molecular weight/number-average molecular weight.

## Data Availability

All data generated or analyzed during this study are included in this published article. The data of the current study are available from the corresponding author upon request.

## Abbreviations

The following abbreviations are used in this manuscript:

ADFI	Average daily feed intake
ADG	Average daily gain
AGPs	Antibiotic growth promoters
BW	Body weight
FCR	Feed conversion ratio
LEfSe	Linear discriminant analysis effect size
MWCO	Molecular weight cutoff
OTUs	Operational taxonomic units
PCA	Principal components analysis
PMP	1-phenyl-3-methyl-5-pyrazolone
SCFAs	Short-chain fatty acids
SRA	Sequence Read Archive
